# Supplementary material for: The High Mobility Group A1 (HMGA1) Chromatin Architectural Factor Modulates Nuclear Stiffness in Breast Cancer Cells
Source: Int J Mol Sci. 2019 Jun 4;20(11):2733. doi: 10.3390/ijms20112733 (PMC6600462; doi:10.3390/ijms20112733)
Supplement: Supplementary file 1 [file ijms-20-02733-s001.pdf]

**Supplementary figures and tables – SENIGAGLIESI et al.**

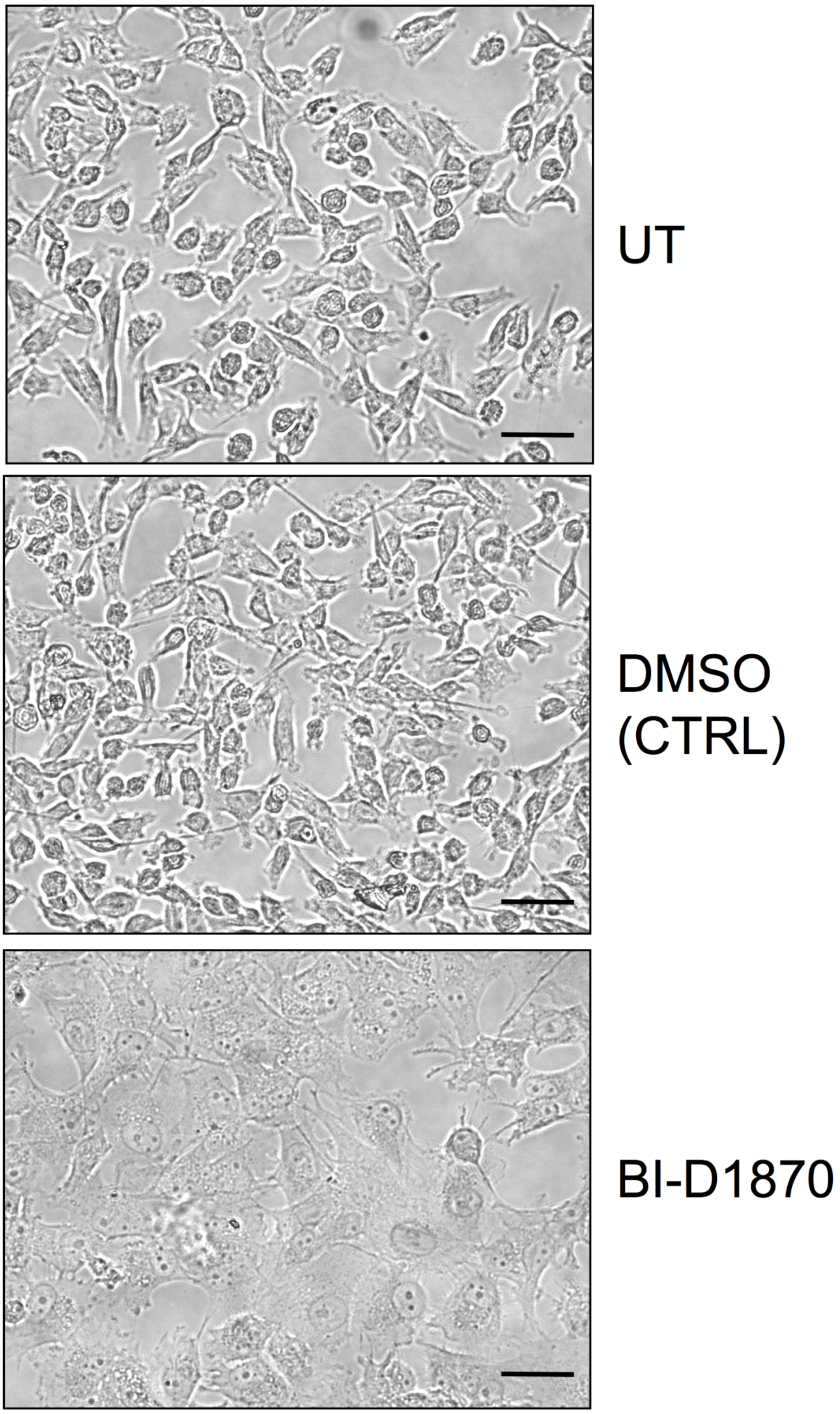


**Supplementary Figure 1. Cells treated with BI–D1870 undergo a morphology transition resembling MET.** Optical images of MDA–MB–231 cells left untreated (UT), treated with DMSO (CTRL, 0.05%), and with BI-D1870 (10 μM – 24h). Scale bars represent 50 μm.

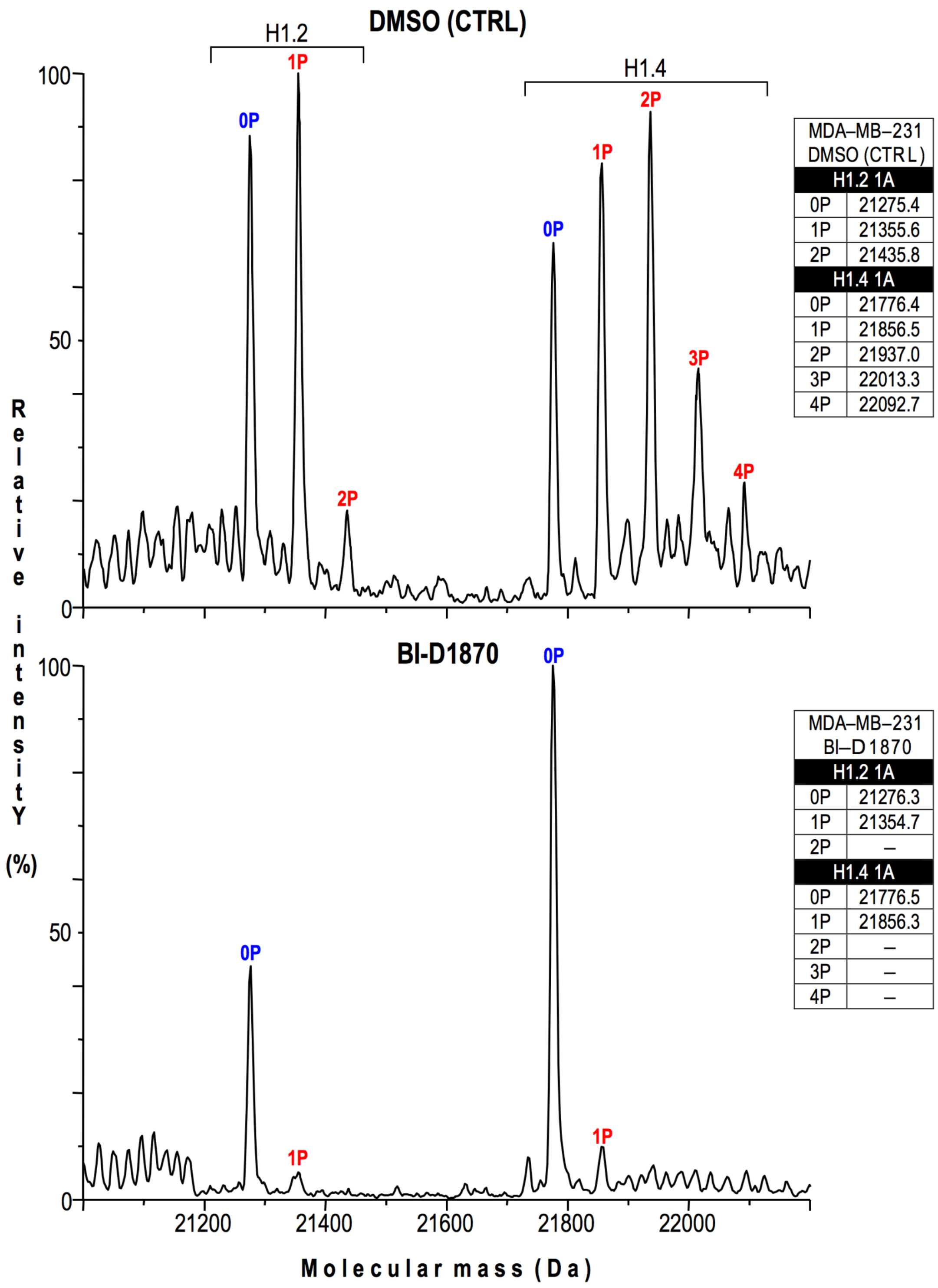


**Supplementary Figure 2: Histone H1 is massively dephosphorylated upon mesenchymal–to–epithelial transition induced by BI–D1870 treatment.** Reconstructed mass spectra of histone H1 variants obtained from MDA–MB–231 cells treated with DMSO (CTRL, 0.05%) and with BI–D1870 (10 μM) for 24 hours. The two histone H1 variants (H1.2 and H1.4) expressed by MDA–MB–231 cells are indicated on the top of the first reconstructed mass spectra. 0P, 1P, 2P, 3P, and 4P indicate the number of phosphate groups. On the right side molecular masses (Da) of the different histone H1 peaks are indicated.

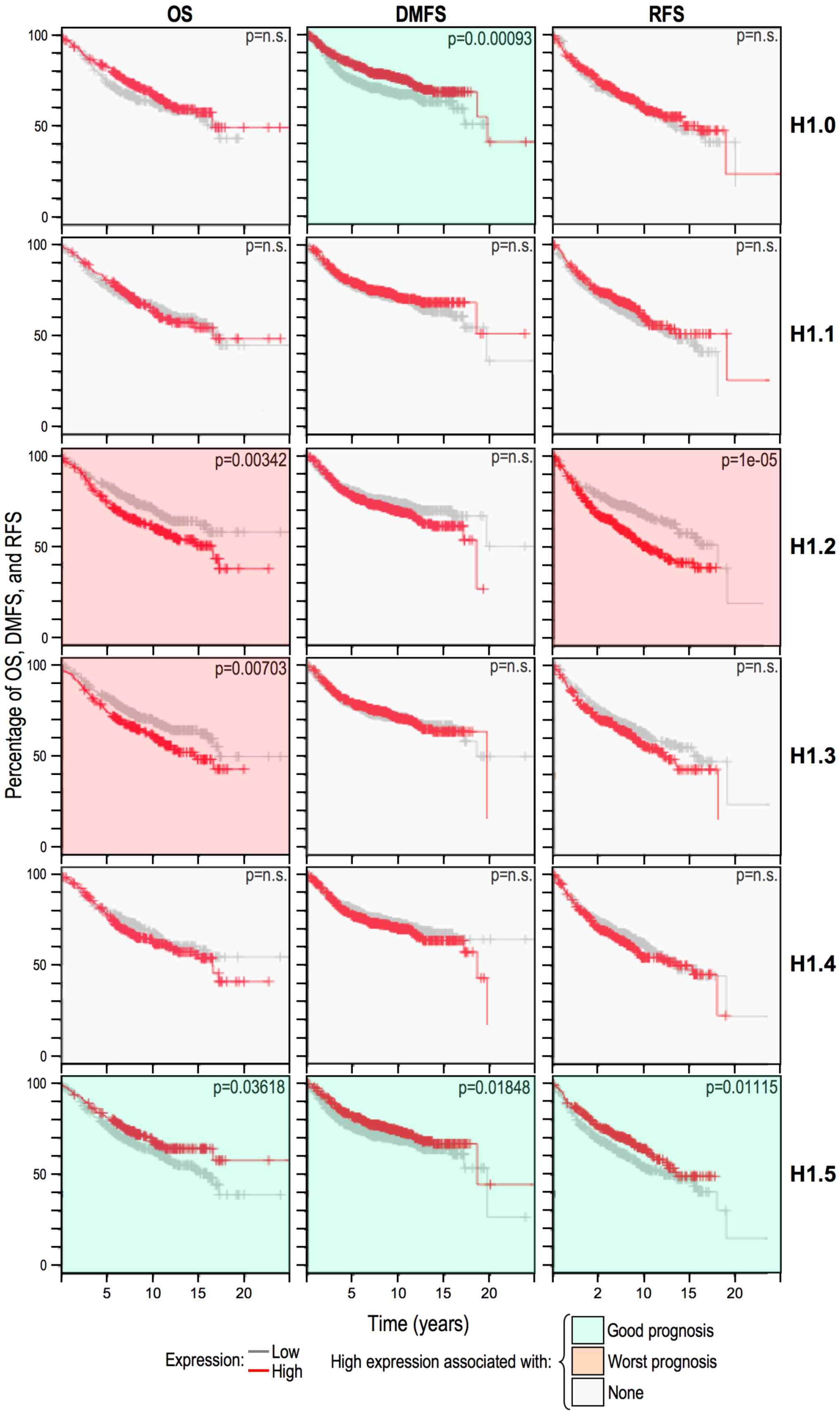


**Supplementary Figure 3: The prognostic value of histone H1 variants gene expression levels in breast cancer specimens.** Kaplan–Meier plots for overall (OS), relapse–free (RFS), and distant–metastasis–free (DMFS) survival with regards to the histone H1.0, H1.1, H1.2, H1.3, H1.4, and H1.5 gene expression level (low or high) using the online tool Gene Expression-Based Outcome for Breast Cancer Online (GOBO, http://co.bmc.lu.se/gobo).

| Supplementary table 1A | | | | |
|---|---|---|---|---|
| **MDA–MB–231 siCTRL** | | | | |
| HMGN2 th. | **HMGN2 exp.** | | **Error (ppm)** | |
| 9261.5 | **9262.1** | | **64.78432219** | |
| Histone H1 isoform | **Exp.** | **Error** | **Adjusted** | **Intensity** |
| H1.2 1A 0P | **21277.8** | **1.4** | **21276.4212** | **2874510** |
| H1.2 1A 1P | **21357.7** | **1.4** | **21356.31602** | **2008087** |
| H1.4 1A 0P | **21778.2** | **1.4** | **21776.78877** | **3607528** |
| H1.4 1A 1P | **21858.6** | **1.4** | **21857.18356** | **2308038** |
| H1.4 1A 2P | **21938.5** | **1.4** | **21937.07839** | **1889553** |
| H1.4 1A 3P | **22017.3** | **1.4** | **22015.87328** | **1070319** |
| **MDA–MB–231 siA1_3** | | | | |
| HMGN2 th. | **HMGN2 exp.** | | **Error (ppm)** | |
| 9261.5 | **9262.5** | | **107.9738703** | |
| Histone H1 isoform | **Exp.** | **Error** | **Adjusted** | **Intensity** |
| H1.2 1A 0P | **21278.1** | **2.3** | **21275.80197** | **3947572** |
| H1.2 1A 1P | **21356.3** | **2.3** | **21353.99352** | **1096102** |
| H1.4 1A 0P | **21778.8** | **2.4** | **21776.44789** | **7637806** |
| H1.4 1A 1P | **21858** | **2.4** | **21855.63934** | **1250661** |
| H1.4 1A 2P | **21942.1** | **2.4** | **21939.73025** | **953907** |
| **MDA-MB-157 siCTRL** | | | | |
| HMGN2 th. | **HMGN2 exp.** | | **Error (ppm)** | |
| 9261.5 | **9261.7** | | **21.59477406** | |
| Histone H1 isoform | **Exp.** | **Error** | **Adjusted** | **Intensity** |
| H1.2 1A 0P | **21277.9** | **0.5** | **21277.43189** | **7373493** |
| H1.2 1A 1P | **21356.8** | **0.5** | **21356.33015** | **2822784** |
| H1.4 1A 0P | **21776.8** | **0.5** | **21776.32091** | **10438161** |
| H1.4 1A 1P | **21857.3** | **0.5** | **21856.81914** | **3324913** |
| H1.4 1A 2P | **21937.9** | **0.5** | **21937.41737** | **2592616** |
| H1.4 1A 3P | **22016.7** | **0.5** | **22016.21563** | **1605214** |
| **MDA–MB–157 siA1_3** | | | | |
| HMGN2 th. | **HMGN2 exp.** | | **Error (ppm)** | |
| 9261.5 | **9262.2** | | **75.58170923** | |
| Histone H1 isoform | **Exp.** | **Error** | **Adjusted** | **Intensity** |
| H1.2 1A 0P | **21277.1** | **1.6** | **21275.48294** | **8136894** |
| H1.2 1A 1P | **21356.1** | **1.6** | **21354.47694** | **2379205** |
| H1.4 1A 0P | **21777.5** | **1.7** | **21775.84491** | **13274109** |
| H1.4 1A 1P | **21857.8** | **1.7** | **21856.13881** | **3090011** |
| H1.4 1A 2P | **21937.4** | **1.7** | **21935.73276** | **2279963** |

**Supplementary table 1B**

**MCF7 CTRL (HA–empty)**

| HMGN2 th. | HMGN2 exp. | | Error (ppm) | |
|---|---|---|---|---|
| 9261.5 | 9264.9 | | 367.1111591 | |
| | | | | |
| Histone H1 isoform | Exp. | Error | Adjusted | Intensity |
| H1.2 1A 0P | 21282.1 | 7.8 | 21274.28947 | 3208750 |
| H1.2* 1A 0P | 21312.8 | 7.8 | 21304.9782 | 2797405 |
| H1.2 1A 1P | 21363.2 | 7.8 | 21355.35971 | 1934787 |
| H1.2* 1A 1P | 21392.8 | 7.9 | 21384.94884 | 1694990 |
| | | | | |
| H1.4 1A 0P | 21784.2 | 8.0 | 21776.2052 | 4824531 |
| H1.4 1A 1P | 21863.5 | 8.0 | 21855.4761 | 3885962 |
| H1.4 1A 2P | 21943.1 | 8.1 | 21935.04688 | 2314448 |
| H1.4 1A 3P | 22023.2 | 8.1 | 22015.11749 | 1754361 |
| | | | | |
| H1.3 1A 0P | 22268 | 8.2 | 22259.82764 | 5896007 |
| H1.3 1A 1P | 22348.1 | 8.2 | 22339.89825 | 1411300 |
| | | | | |
| H1.5 1A 0P | 22497.4 | 8.3 | 22489.14345 | 9484286 |
| H1.5 1A 1P | 22578.7 | 8.3 | 22570.41362 | 6058861 |
| H1.5 1A 2P | 22658.2 | 8.3 | 22649.88444 | 4894543 |
| H1.5 1A 3P | 22739 | 8.3 | 22730.65479 | 2226158 |
| H1.5 1A 4P | 22818.8 | 8.4 | 22810.4255 | 701619 |

**MCF7 HA–A1**

| HMGN2 th. | HMGN2 exp. | | Error (ppm) | |
|---|---|---|---|---|
| 9261.5 | 9265.9 | | 475.0850294 | |
| | | | | |
| Histone H1 isoform | Exp. | Error | Adjusted | Intensity |
| H1.2 1A 0P | 21283.7 | 10.1 | 21273.59024 | 1302106 |
| H1.2* 1A 0P | 21312.4 | 10.1 | 21302.27661 | 1200364 |
| H1.2 1A 1P | 21362.7 | 10.1 | 21352.55272 | 1226233 |
| H1.2* 1A 1P | 21394.9 | 10.2 | 21384.73742 | 1103658 |
| | | | | |
| H1.4 1A 0P | 21784.1 | 10.3 | 21773.75255 | 2060259 |
| H1.4 1A 1P | 21863 | 10.4 | 21852.61508 | 2269417 |
| H1.4 1A 2P | 21944 | 10.4 | 21933.5766 | 1714262 |
| H1.4 1A 3P | 22022.6 | 10.5 | 22012.13927 | 934264 |
| | | | | |
| H1.3 1A 0P | 22269.1 | 10.6 | 22258.52218 | 2537899 |
| H1.3 1A 1P | 22348.1 | 10.6 | 22337.48465 | 957037 |
| H1.3 1A 2P | 22429.2 | 10.7 | 22418.54613 | 718465 |
| | | | | |
| H1.5 1A 0P | 22497.6 | 10.7 | 22486.91364 | 3868668 |
| H1.5 1A 1P | 22578.8 | 10.7 | 22568.07507 | 5155595 |
| H1.5 1A 2P | 22660 | 10.8 | 22649.2365 | 4366874 |
| H1.5 1A 3P | 22738.8 | 10.8 | 22727.99907 | 2031679 |
| **H1.5 1A 4P** | 22821.6 | 10.8 | 22810.75974 | 690993 |
